# Critical assessment of health financing indicators and expenditure in South Africa

**DOI:** 10.4102/hsag.v31i0.3142

**Published:** 2026-01-27

**Authors:** Njabulo I. Mkhize

**Affiliations:** 1 Department of Economics, College of Economic and Management Sciences, University of South Africa, Pretoria, South Africa

**Keywords:** public health care, expenditure, health financing, South Africa, diseases

## Abstract

**Background:**

Despite the increased funding and advancements in providing public health services, the health system continues to be challenged by the high prevalence of diseases.

**Aim:**

The objective of this study is to review health sector financing in South Africa by highlighting causes for spending pressures in the health system. The study setting comprised the National Department of Health in Pretoria.

**Method:**

The study employs a qualitative approach by reviewing the literature on healthcare financing and expenditure in South Africa through the analysis of multiple electronic database searches to provide insight into trends and behaviour of health care budgets, expenditure, and health financing indicators.

**Results:**

Health financing indicators reveal that South Africa’s real health budgets have increased over the past years. However, the increase has barely kept pace with population growth rates. Also, the analysis of the consumer price index (CPI) and medical inflation shows that the costs for medicines and other consumables have, over time, remained significantly above the normal consumer inflation.

**Conclusion:**

The rising trend in population growth and life expectancy rates suggests that health care funding will need to rise considerably in the next decade as individuals live longer and are anticipated to be given unrestricted access to public health services.

**Contribution:**

This study deepens the comprehension that, while reforms in the health sector and mobilisation of additional resources are essential for tackling disparities and access to health care, it is also crucial to examine and improve the efficiencies in the utilisation of these resources.

## Introduction

A study by Mkhize (2017) indicates that there is a widespread belief that health expenses will rise considerably over the next decade as individuals live longer and expect to be given unrestricted access to public health care. This indicates that any formal structure or system for providing health care will eventually be confronted with the unavoidable challenge of a limited amount of resources to cater for an expanding patient population (Zere, McIntyre & Addison 2001). In South Africa, spending on public health care represents approximately 4% of gross domestic product (GDP) and supports a rising number of medically uninsured persons. South Africa has a quadruple disease load, with chronic illnesses, trauma, and diseases of development like infectious diseases coexisting (National Treasury 2009).

Despite the rising allocations and progress made with the delivery of public health services, the health system continues to be challenged by the large burden of diseases, especially from HIV and tuberculosis (TB), not being adequately prevented (National Treasury 2009). Burden of diseases puts enormous pressure on provincial health budgets and raises concerns around the financial instability and capacity of provinces to cope. The current equitable share formula does not make an explicit inclusion of the provinces’ burden of disease factor in determining provincial health allocations, and hence does not adequately respond to the health needs. Since the early 1990s, a number of South African health care researchers and analysts have made calls for a needs-based formula to redistribute health resources more equitably (Bourne et al. 1990).

In February 2000, the Financial and Fiscal Commission made recommendations for a new approach in the allocation of national revenues based on national norms and standards for the provision of basic services in the health sector. It was hoped that the new approach would provide provinces with the sufficient resources needed to render health services, as it attempted to estimate the costs of providing the most basic health services across provinces. Moreover, it was argued that the proposed formula could be a response to endeavours for a long-term approach to the equitable share allocation for health.

Critics of the current provincial equitable share formula argue that poor provinces have less spending autonomy, given the fact that the current formula rewards provinces according to economic output and hence poor provinces tend to rely more heavily on conditional grants to address their high burden of diseases (McIntyre, Muirhead & Gilson 2002). In addition, in the current system, it has been found that there has been a substantial increase in private sector investment in health care in wealthier provinces as more resources are distributed to rich provinces with better health infrastructure (Shevel 2002). Unlike the current system, in the costed norms approach, costs (or allocations) are influenced by the degree of ruralness in a province, the incidence of poverty and various disease profiles. This makes the costed norms approach an appropriate mechanism for achieving greater equity and improved performance for health care in South Africa. The objective of this study is to review health sector financing in South Africa, by highlighting causes for spending pressures in the health system as well as the factors that are likely to influence and undermine an efficient allocation of health resources. This research is anticipated to contribute to the understanding of healthcare financing and spending pressures in the health system, ultimately leading to more efficient allocation of health resources.

## Research methods and design

The study reviews the literature on healthcare financing and expenditure in South Africa by analysing multiple electronic database searches to establish the flow of funding in the overall health system. It utilises secondary data to provide insight into trends and behaviour of health care budgets, expenditure, health financing indicators, and health outcomes.

The literature review process employed in this study includes searching extant literature from various academic databases, including Scopus and JSTOR, for international journals as well as SABINET local South African publications, using keywords including ‘public health care’, ‘expenditure’, and ‘health financing’. The use of double quotation marks is to ensure that the keywords are searched as exact phrases. Synthesised findings are integrated, and conclusions are drawn to provide a comprehensive overview of the research focus based on this all-inclusive knowledge base.

## Adequacy of public health care funding in South Africa

**FIGURE 1:**
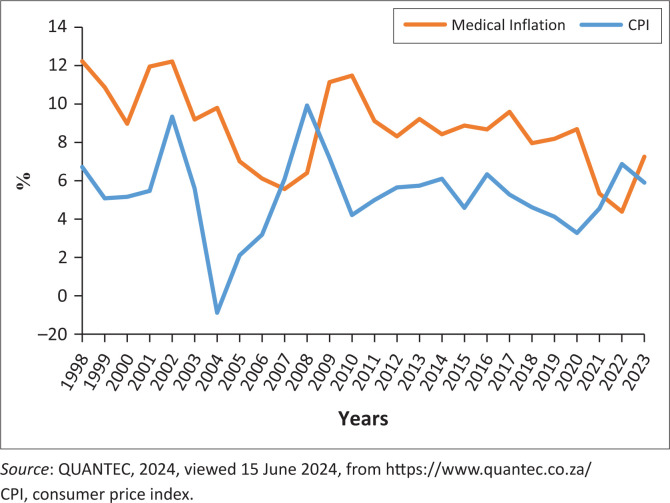
Consumer price index versus medical inflation.

The operational costs of rendering health care services are rising more quickly than the overall allocations. This is partly because of the escalating costs of medicines and medical consumables, as well as the lagged effects of the centralised wage agreements and their impact on the overall wage bill (and ultimately unit cost of staffing), which subsequently overstretch health budgets. Figure 1 shows an analysis of consumer price index (CPI) inflation versus medical inflation in South Africa.

Medical inflation, which is used to estimate the escalating costs for medicines and other consumables, has over time remained significantly above the normal consumer inflation. Between 2010 and 2023, medical inflation has ranged between 11.1% and 7.3%, with an average of 8.2%. During the same period, the headline CPI ranged between a high 7.2% and low 5.9%, with an average of 5.2%. Hence, average medical inflation had remained above average CPI by 3.0% over this period. In December 2023, medical inflation figures as calculated by Statistics South Africa (STATSSA) came in at 6.8%. This pattern has been observed in most parts of the world; for instance, in the United States (US), the difference between medical and manufactured goods inflation has been a consistent 2.6% points a year since 1968. In South Africa, it has been suggested that the main drivers in this regard have been the ageing population, high electricity tariff increases, laboratory services, blood and blood products, medical technology, costs of medicines and waste.

As with most other countries, South Africa has improved technologies that enable more sophisticated treatment. Minimally invasive surgeries and more sophisticated scanning technologies allow patients to be treated for a shorter period with better outcomes. This has implications for the cost of treatment. That is, technology tends to increase the cost per patient treated, given a patient is treated within a short space of time. All of these, and other factors, exert a steady upward pressure on the overall costs of rendering health care services.

During its preparation of expenditure estimates for the Medium Term Expenditure Framework (MTEF), the National Treasury has consistently emphasised that the CPI assumptions or inflationary adjustments are used to generate the outer years (budgets) of the MTEF period. For instance, in planning for the 2024 MTEF budgets, provinces had to adhere to Treasury’s projections for headline CPI inflation, which are:

- 2024/25 financial year: 4.8%.
- 2025/26 financial year: 4.7%.
- 2026/27 financial year: 4.6%.

While these assumptions may be useful in informing the provisions for general price increases and hence the budget allocations, CPI cannot better contain the cost of health care without reducing the quality of care. In the US, policymakers have now chosen to utilise medical inflation as a standard for health care budgeting rather than the traditionally low consumer price inflation, which has consistently been lower than medical inflation (Mkhize 2017).

## Health financing indicators

Despite the increases in domestic and external assistance for health, over the past decade, health resources in most low-income and middle-income countries are still not sufficient to ensure a universal coverage of basic needed interventions. While the magnitude of how much is exactly needed is debatable, the adjustment estimates of the Commission on Macroeconomics and Health revealed a need of around US$40 per person per year (World Health Organization [WHO] 2008).

Health financing is fundamental towards ensuring that the health system has the ability to maintain and improve the welfare of the population. It is concerned with the mobilisation, accumulation and allocation of money to cover the health needs of the people in the health system as well as to ensure that, with the funding available, people have access to effective public health and personal health care (World Health Report 2000). Hence, the health financing indicators capture the extent to which people are protected from financial risks associated with ill health. These indicators provide an understanding of the patterns of investment, expenditure, sources of funding and proportions of total allocation.

**FIGURE 2:**
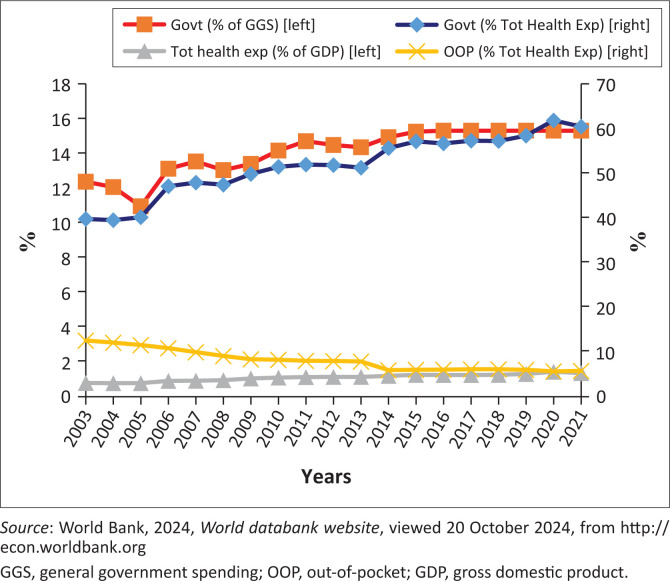
Health financing indicators.

Over the past years, a number of sectoral reviews have been undertaken to evaluate the progress and performance of the health sector. Recent data on the flow of funds in the health system show that approximately 40% of total health care funds in South Africa flows from public sector financing intermediaries, that is, national, provincial and local departments of health, while 60% flows via private intermediaries (Day & Gray 2005). The largest contributor being medical schemes which account for about 46% of health care expenditure, followed by provincial departments of health, which make up 38%. The contribution by households, in the form of out-of-pocket payments paid directly to health care providers, amounted to approximately 14% of all health care expenditure. Figure 2 shows health financing indicators. The government’s health expenditure as a percentage of total health expenditure remained on a steadily upward path. In the 2020/21 main budget, the government’s health expenditure was recorded at around 60% of total health expenditure and 15% of general government spending (GGS).

According to the Abuja Declaration of 2001, the African heads of state committed to ensuring that 15% of the overall government’s expenditure goes to health (WHO 2008). Hence, the current level of 15% could be viewed as a reflection of the government’s sustained level of commitment.

According to the WHO (2008) report on health systems financing, the general consensus for health financing systems should not only seek to raise sufficient funds for health, but should do so in a way that allows people to use the needed service without the risk of severe financial hardship or impoverishment. The World Bank has defined a financial catastrophe as occurring when out-of-pocket (OOP) expenditure exceeds 10% of a household’s total income.

In South Africa, while it is true that private health care consumes over 50% of total health care spending, not all financing of private health care stems from medical schemes. A significant share of out-of-pocket spending goes to private health care, particularly in poor communities. Spending on health care as a proportion of family income doubled in black households since the first half of the 1990s, and in the lowest income categories, less than one-fifth of private health care consultations are covered by insurance (Söderlund, Schierhout & Van den Heever 1998). This is a clear reflection of inequities in the distribution of public health care to the impoverished.

**FIGURE 3:**
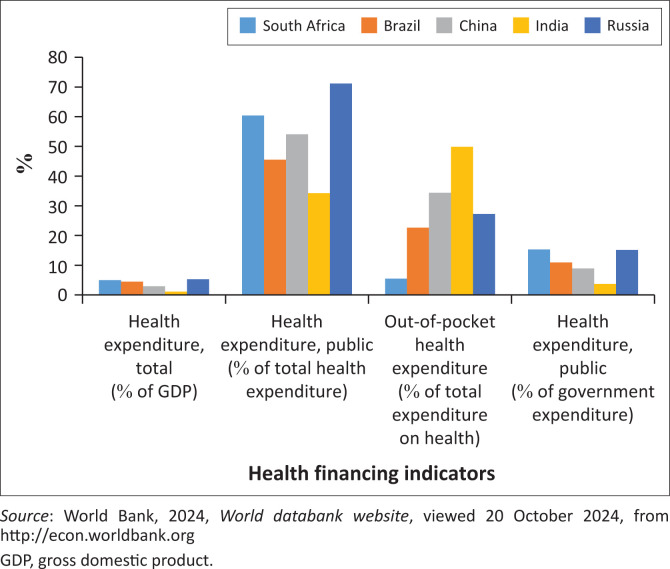
Health financing indicators among BRICS countries.

Figure 3 shows health sector financing indicators across BRICS countries. As can be seen, based on international comparison data regularly released by the World Bank, South Africa is on par with similar middle-income countries. However, past analyses on real health budgets reveal that it has barely kept pace with population growth rates (Söderlund et al. 1998). In the public sector, health care expenditure has been relatively stagnant in real per capita terms over the past years (McIntyre & Thiede 2007). Hence, unless alternative sources of health care funding are identified, all resource redistribution will have to be met within the existing fiscal envelope.

## Causes for spending pressures in the health system

One of the key causes of spending pressures in the health system relates to the supply of health care. Appleby (2013) reported that technological change (such as new medicines and new surgical techniques) has been identified as a dominant factor that has generally accounted for more health spending over the past years. That is, medical technology has not only expanded the range and scope of what is possible in health care, but has also led to higher spending.

Furthermore, the increases in costs have also been significant, considerably reducing the gains from the new technology. In South Africa, the costs for medicines and other consumables have, over time, remained significantly above the normal consumer inflation, and this has serious implications for the cost of treatment and resource allocation.

On the demand side, increases in the overall population size, high burden of disease and the proportion of high users of care, such as older people, have led to increased spending in the health care system. Analyses of real health budgets reveal that they have barely kept pace with population growth rates (Söderlund et al. 1998). According to Stuckler et al. (2011), the general measures of burden of disease (i.e. HIV prevalence, infant mortality rates, and crude total death rates) explain roughly one-fifth of the variation in health spending among the South African provinces from 2001 to 2007. Epidemiologists maintain that South Africa suffers from a quadruple burden of disease (Mkhize 2017). Diseases of development, such as communicable diseases, co-exist with a growing problem of chronic illnesses and trauma (National Treasury 2009).

## Conclusion

Despite the increased funding and advancements in providing public health services, the health system continues to be challenged by the high prevalence of diseases. South Africa faces a fourfold burden of disease where diseases of development, such as communicable diseases, co-exist with the growing problem of chronic diseases and trauma (Mkhize 2017).

There are increasing concerns that public health is utilising more resources without a corresponding rise in the output of service provision, thereby raising doubts about its technical efficiency. In South Africa, significant focus from policy makers, donors and health care researchers has been directed towards health sector reform and the mobilisation of additional resources to tackle inequity and improve access to health care. Nonetheless, it is equally crucial to examine and tackle the efficiency with which these resources are utilised.

Moreover, although there have been real increases in health budgets recently, the rising trend in life expectancy indicates that health care funding will need to grow substantially over the coming decade as people live longer and anticipate unrestricted access to public health services. If maintained, this could possibly alter the demographic profile of South Africa, impacting how public health is financed.

Lastly, the analysis of CPI and medical inflation also shows that the costs for medicines and other consumables have, over time, remained significantly above the normal consumer inflation. This has serious implications for the cost of treatment and resource allocation.
